# Evaluating usability, adherence and clinical benefit of a new digital heath application in rheumatoid arthritis: a pilot feasibility study

**DOI:** 10.1007/s00296-025-06060-6

**Published:** 2026-01-10

**Authors:** Valerie Schlaht, Thomas Lenzen, Tim Fellerhoff, Magdalena Binder, Anna-Maria Liphardt, Sebastian Rudolf, Paloma Palm von Alten Blaskowitz, Georg Schett, Harriet Morf

**Affiliations:** 1 Department of Internal Medicine 3-Rheumatology & Immunology, Universitätsklinikum Erlangen, Friedrich-Alexander-Universität (FAU) Erlangen-Nürnberg, 18 Erlangen, 91054 Erlangen, Germany; 2 Deutsches Zentrum Immuntherapie, Universitätsklinikum Erlangen, FAU Erlangen-Nürnberg, Erlangen, Germany; 3 Fimo Health GmbH, Bonn, Germany

**Keywords:** Patient education, Rheumatoid arthritis, Digital application, Digital therapeutics, Quality of life

## Abstract

Digital health interventions hold promise for improving self-management in chronic inflammatory diseases such as rheumatoid arthritis (RA). The Fimo Health App was developed to support patients with RA using a multidimensional approach combining educational modules, symptom tracking, and mind–body exercises. This pilot feasibility study aimed to evaluate the usability, adherence, and potential clinical benefit of a prototype version of the app in patients with RA. A further objective was to explore patient subgroups that may particularly gain from the intervention. In a single-arm pilot trial, 31 patients with RA used the app over 12 weeks. Usability was assessed via the System Usability Scale (SUS). Primary clinical outcomes were patient-reported quality of life (SF-36 mental and SF-36 physical = Short-Form 36 Questionnaire), fatigue (FACIT-F), and pain (Visual Rating Scale Pain = VRS Pain), Secondary outcomes included functional impairment (HAQ = Health Assessment Questionnaire), global assessment, fear of motion (TSK = Tampa Scale of Kinesiophobia) and physical activity related-health competency (PAHCO). Subgroup analyses were conducted post hoc based on clinical characteristics. Usability was high (SUS = 74.4) and engagement with core app features strong (97% any use; 70% exercise completion). Across the full cohort (n = 31), changes in the primary outcomes were small overall. Physical quality of life (SF-36) showed a within-person median increase at 6 months (Hodges–Lehmann Δ =  + 5.3; r = 0.50), while fatigue (FACIT-F) and pain (VRS) exhibited only small, non-significant numerical improvements (Δ ≈ + 1 to + 4; r ≈ 0.1–0.2). Mental quality of life (SF-36 mental) remained largely stable. In the subgroup of recently diagnosed patients (≤ 2 years; n = 12), trends were more pronounced. Physical quality of life (SF-36 physical) improved consistently at 3 and 6 months (Δ =  + 1.3 to + 8.7; *r* = 0.67–0.85), accompanied by small favorable shifts in fatigue (Δ ≈ –1.5 to + 2; *r* ≈ 0.2–0.3). Mental quality of life again (SF-36 mental) remained stable over time. The Fimo Health App is a usable and well-accepted digital intervention for patients with RA. Early diagnosed Patients showed signals of potential benefit.. These findings support the value of targeted digital tools early in the RA disease trajectory. *Trial Registration number*: DRKS00035194, registration date: 01.10.2024, https://drks.de/register/de/trial/DRKS00035194/preview.

**Supplementary Information:**

The online version contains supplementary material available at 10.1007/s00296-025-06060-6.

## Introduction

Rheumatoid Arthritis (RA) is a chronic, inflammatory autoimmune disease with a global prevalence of 0.5%, leading to joint destruction and functional disability if it is not adequately controlled [1, 2]. Its symptoms, including pain, swelling, and stiffness, significantly impair physical function, daily activities, and social participation [3, 4]. Beyond articular manifestations, RA can impact other organs, with cardiovascular and interstitial lung diseases representing severe systemic involvement [1, 2]. These multifaceted consequences highlight the profound impact of RA on patients' life. Studies consistently show that high disease activity, lower functional status, pain, and psychological distress (depression and anxiety) are significantly associated with reduced health-related quality of life (QoL) in RA patients [5, 6]. Furthermore, factors like living alone, lower educational level, chronic comorbidities, and radiological damage are linked to poorer QoL, emphasizing the need for comprehensive clinical management addressing both physical and mental aspects of the disease to improve long-term patient outcomes [7–9]. Salaffi et al. showed that patients with RA, psoriatic arthritis (PsA) and axial spondyloarthritis (axSpA), exhibited significantly lower QoL scores compared to healthy individuals. All three diseases demonstrated significant limitations across all 8 scales of the SF-36, including both physical and mental component summaries, compared to the healthy control group.

Fatigue and depressive moods profoundly impact the QoL. RA patients experience fatigue more often compared to healthy subjects. In a study from Spain, fatigue was closely connected to inflammation markers and disease activity in patients with RA [10]. A recent publication showed that there was an indirect positive effect of fatigue on depressive symptoms through self-efficacy. The authors recommended interventions that extend from the management of fatigue and further incorporate the improvement of self-efficacy sense into the RA therapy [11]. EULAR recommendations for treating fatigue provides crucial guidance for health professionals, emphasizing a biopsychosocial approach, shared decision-making, and tailored interventions, including physical activity and psychoeducation, alongside routine fatigue assessment and consideration of disease activity [12].

Physical activity is a key intervention to improve fatigue and depressive disorder. The EULAR guidelines for physical activity in inflammatory diseases recommend physical activity for all rheumatic diseases. General physical activity guidelines, encompassing cardiorespiratory fitness, muscle strength, flexibility, and neuromotor performance, are applicable, feasible, and safe for people with rheumatic diseases. It is always recommended to be physically active, regardless of age, physical limitations or fitness level [13]. In a systematic review, 23 studies were analyzed to investigate the effects of physical activity, meditation and mindfulness on RA patients. The interventions demonstrated various benefits, particularly improving patient-reported outcomes like vitality and mental health, and reducing subjective disease activity parameters such as pain and morning stiffness. Patients with comorbid depressive disorders particularly benefit, as evidenced by a reduction in antidepressant medication [14]. Interestingly, physical activity not only improves physical function, but also cognitive impairments [15]. In a randomized clinical trial, patients with RA improved in cognitive function and fatigue after exercising [15].

Digital technologies can be an advantage in movement therapy. Many patients want to be flexible and be able to train at any time and in any place. However, in a 2020 survey by Knitza et al., the majority of patients with rheumatic diseases stated that they knew little about digital applications in rheumatology, even though they widely use the internet for medical searches [16]. Although Germany is a pioneer of digital technologies in medicine, especially through the digital law and the introduction of digital health applications (DHA) in diagnostics and therapy, only a few digital applications exist in rheumatology [17]. So far, there are digital applications for supporting diagnostics in form of symptom checker such as *Ada Health* or *RhePort*. For complementary therapy, the patient organization *Rheuma Liga* has developed the RheumaAuszeit application, which focuses primarily on physical exercises and relaxation and has little educational content [18]. In the “*Axia app*” for spondyloarthritis, patients can choose from over 100 physiotherapy exercises specifically for the underlying disease and practise them at home. The app is currently still being tested in SpA patients [19].

### Description of the Fimo Health App

The Fimo Health App is a CE-certified digital health application designed to support patients with chronic inflammatory and fatigue-related conditions in self-managing their disease [20]. In this study, participants used the RA-specific final version (v2.0) of the app. The intervention combines daily symptom monitoring, educational modules, and evidence-based exercises delivered through a mobile interface.

Educational content is structured into 42 short, interactive modules addressing topics such as fatigue management, physical activity, pain, stress reduction, sleep quality, social support and pacing strategies. Modules are unlocked sequentially and contain multimedia elements (videos, quizzes, and short reflective exercises) developed in collaboration with clinicians, psychologists, and physiotherapists.

Exercise components include guided physical activity routines (mobility, strength, relaxation, and breathing exercises), designed for home use without equipment. The exercises are adapted in difficulty and frequency based on self-reported fatigue levels and daily symptom data.

Symptom tracking is performed through daily and monthly check-ins assessing e. g. symptoms and severity, sleep quality, and activity levels. Data entry is user-initiated but prompted by reminders within the app.

Feedback and visualization are provided through interactive dashboards that display symptom trends, correlations (e.g., pain vs. sleep), and personal progress over time. Users receive automated motivational feedback and contextualized suggestions (e.g., “Consider a rest today” when fatigue levels are high).

**Fig. 1:**
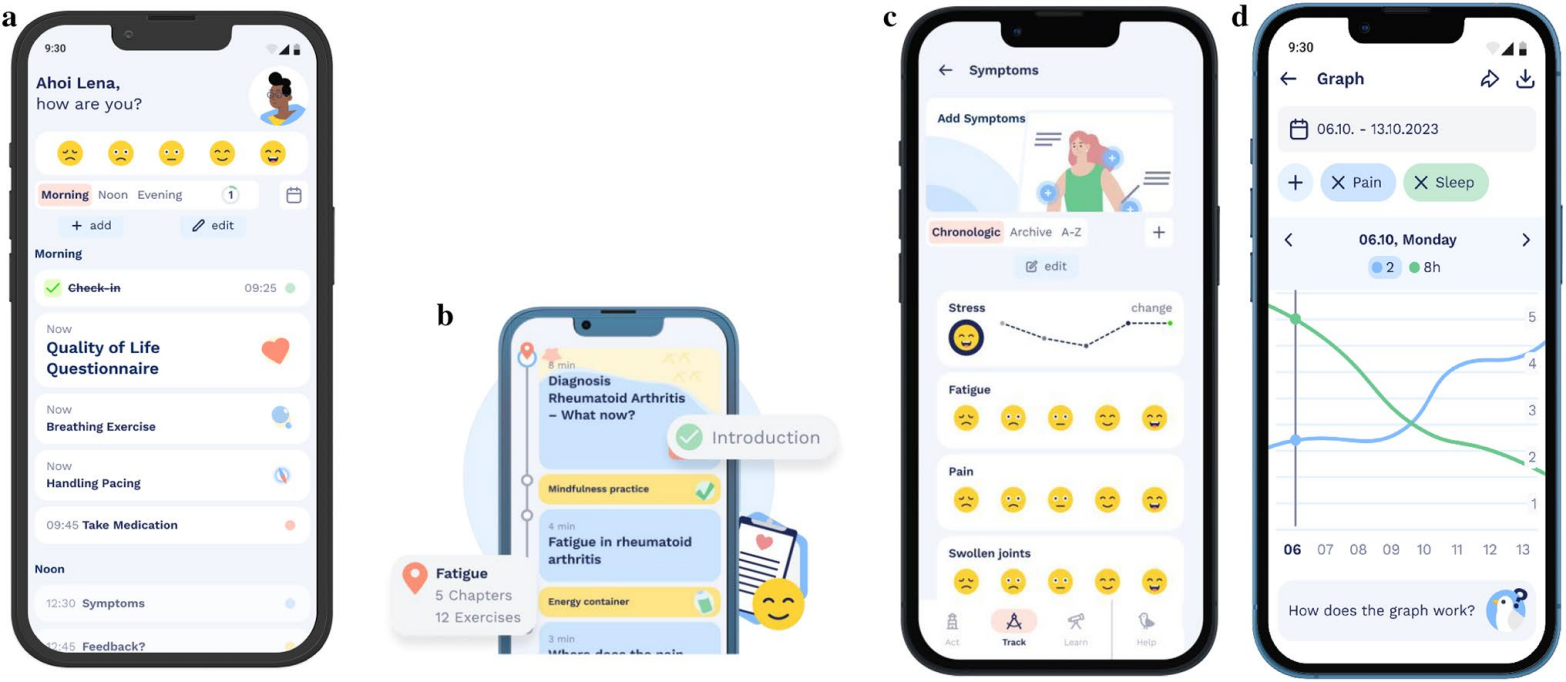
**a** Design and user interface of the Fimo Health App for rheumatoid arthritis (RA): Home Screen. **b** Design and user interface of the Fimo Health App for rheumatoid arthritis (RA): example module layout. **c** Design and user interface of the Fimo Health App for rheumatoid arthritis (RA): Symptom Tracking. **d** Design and user interface of the Fimo Health App for rheumatoid arthritis (RA): User Insights

Usage and adherence data are collected automatically via in-app telemetry (e.g., number of active days, average use, average app openings per week). A visual overview of the app interface and functionality is provided in Fig. 1a-d, illustrating home screen, an example module layout, symptom tracking and data feedback.

. The present work was designed as an exploratory mixed-methods feasibility study primarily assessing usability, adherence, and feasibility of data collection, while exploring potential trends in patient-reported outcomes.

## Methods

### Study design and patient recruitment

We conducted a prospective, single-arm, exploratory trial. Patients or the public were not involved in the design, or conduct, or reporting, or dissemination plans of our research.

Patients were recruited between October 2024 and March 2025 from the outpatient clinics of the Department of Rheumatology and Immunology at the university hospital Erlangen, Germany. Potentially eligible patients were first identified by searching the clinic´s internal patient database and pre-screened for clear ineligibility. These patients were contacted by phone to inform them about the study and assess their initial interest in participation. Patients expressing interest underwent a detailed assessment of the inclusion and exclusion criteria, and written informed consent was obtained prior to study participation. Eligible patients were then formally enrolled in the study. End of study was July 2025.

Inclusion criteria were patients with RA (diagnosed according to ACR/EULAR criteria), between 18 and 70 years of age, a first diagnosis that is as recent as possible (in the last 1–5 years) and, no change of medication in the last 3 months [21]. The exclusion criteria included pregnancy, cognitive impairment and the lack of access to a smartphone or inability to use it properly.

### Ethical considerations

The study was approved by the medical faculty ethics committee of the Friedrich-Alexander-Universität Erlangen-Nürnberg, Erlangen, Germany (Friedrich-Alexander University of Erlangen-Nürnberg (FAU), Number: 24–220-Bm, Date: 02.07.2024) and prospectively registered in the German Clinical Trials Register (DRKS-ID DRKS00035194, DRKS—Deutsches Register Klinischer Studien, 01.10.2024). The study was conducted in accordance with the ethical guidelines of the Declaration of Helsinki.

### Data storage

All patient data was pseudonymized. Collected data was stored and analyzed in a password-protected database (REDCap) to which only previously defined and authorized persons had access [22].

### Measurements

The total duration of the study was six months, divided into three assessment points: baseline (week 0), follow-up 1 (week 12; primary analysis), and follow-up 2 (week 24; secondary analysis). After successful inclusion, participants received an email with the first set of standardized questionnaires and an individual activation code for the Fimo Health App (version 2.0). From that point onwards, participants used the app independently with a recommended daily usage time of 10–15 min (Fig. 1). Structured telephone check-ins were conducted after 1, 4, and 8 weeks to monitor engagement, clarify technical issues, and collect qualitative feedback.

A timeline depicting the study´s scheduled time points can be found in the Supplementary Material (Supplement Fig. 2).

### App usage and adherence

App usage and adherence were assessed using a combination of objective telemetry data and structured self-reports to ensure a comprehensive and consistent evaluation.

Objective telemetry data were automatically recorded by the app and analyzed for three key indicators:

- Active days of app use (up to follow-up 1),
- Average usage time per week (minutes), and
- Average number of app openings per week.

Subjective adherence was evaluated through structured telephone interviews at weeks 1, 4, and 8, focusing on perceived regularity of use (e.g., “Do you still use the app?”), engagement with course content, and barriers to continued participation.

### Patient-reported outcomes

Patient-reported outcomes (PROs) were pre-specified in the study protocol (DRKS00035194) and collected via REDCap at baseline, week 12, and week 24. All PROs are shown in Table 1 supplements.

**Table 1:** Baseline demographic and disease-specific characteristics

Patient characteristics (N° = 36)	Mean ± SD/Percent
Age (years), mean ± SD	48.6 ± 17.1
Female	77%
Disease Duration (years), mean ± SD	5.3 ± 6.1
DMARD intake (overall)	87%
Conventional synthetic DMARD	68%
Targeted synthetic DMARD	13%
Biologic DMARD	16%
Glucocorticoid intake	3%

Furthermore, app usability was evaluated using the SUS questionnaire (system usability scale) and the Mobile App Rating Scale (MARS).

All data were collected via patient reported outcomes.

All questionnaires have been previously validated and are widely used in rheumatology and digital health research [29-37].

A detailed overview of the measures, domains, and scoring is provided in Supplementary Table S1.

### Statistical analysis

This study was conducted to generate data for sample size calculations in preparation for a future confirmatory study, thereby increasing the likelihood of a successful main trial. Published guidelines recommend a range of 12 to 50 patients per group for pilot studies [23, 24]. Accordingly, the pilot was not designed to have sufficient statistical power for traditional inferential statistical analysis (including significance testing). We assumed small-to-moderate within-person effects (r ≈ 0.3–0.5) on key patient-reported outcomes such as fatigue and physical quality of life, based on previous digital self-management interventions in rheumatology.

### Data distribution and rationale for non-parametric analysis

All statistical analyses were performed using Python 3.12 (pandas 2.2.3, NumPy 2.1.3, SciPy 1.14.1, Matplotlib 3.9.2). [27, 28].

Prior to comparative analysis, data distributions were assessed using Shapiro–Wilk tests and visual inspection of Q–Q plots. While several outcome variables in the overall cohort approximated normality, this assumption was not consistently met across all endpoints, and deviations were more pronounced within the smaller subgroup of recently diagnosed patients (≤ 2 years).

To ensure methodological consistency and robustness across all outcomes and subgroups, and to avoid bias from outliers or skewed data distributions a non-parametric analysis strategy was adopted for both descriptive and comparative statistics.

Continuous variables are reported as median [interquartile range (IQR)].

### Comparative analysis

Within-subject changes from baseline to 3-month and 6-month follow-up were evaluated using the Wilcoxon signed-rank test, which is suitable for paired, non-normally distributed data [25]. For each comparison, the Hodges–Lehmann estimator was used to quantify the median change (pseudomedian) together with 95% confidence intervals (CIs), providing a measure of the typical within-person change. Confidence intervals were derived using scipy.stats.wilcoxon’s built-in algorithm, which automatically selects exact or asymptotic methods depending on sample size.

Effect sizes were expressed as Wilcoxon’s r = Z / √N, where *Z* is the standardized test statistic and *N* the number of non-zero paired differences. Following Cohen’s conventions, r ≈ 0.1, 0.3, 0.5 were interpreted as small, medium, and large effects, respectively.[26].

Results are reported as Hodges–Lehmann median change [95% CI] and effect size r, emphasizing the magnitude and direction of within-person change rather than statistical significance. P-values are presented solely as descriptive indicators without correction for multiple testing and without inferential interpretation, given the exploratory nature of this pilot study. Missing values were handled pairwise; participants with < 3 non-zero pairs were excluded from Wilcoxon testing, but retained in descriptive summaries.

An additional aim of the study was to identify patient subgroups that particularly show signals of potential benefit from the intervention and may warrant targeted inclusion in future trials. To this end, exploratory post hoc subgroup analyses were conducted based on age, sex, and disease duration. The goal was to explore potential moderators of treatment response to inform both the design of future studies and the further development of the app and its content.

## Results

### Patient characteristics

A total of 36 patients were included in the study, with 5 dropping out during the course of the study, resulting in 31 per-protocol completers at follow-up 1 (12 weeks). No notable differences were observed between participants who discontinued and those who completed the study in terms of baseline characteristics. All 31 participants who completed the 12-week assessment also completed the 24-week follow-up; no additional dropouts occurred between 12 and 24 weeks. Here, we report a complete case analysis.

An overview of the patient flow throughout the enrollment process is provided in the supplements (Supplementary Figure S2). Demographic and disease specific characteristics are shown in Table 1.

### Adherence and usability

Objective telemetry data and self-reported adherence showed consistent patterns, confirming sustained engagement during the active intervention period. Reported adherence rates were high across core app components: 97% of participants actively used the app in general, 85% regularly engaged with the daily schedule, 68% with symptom tracking, 43% with the educational course modules, and 70% with the integrated exercises. During the 12 Week intervention phase, users had around 50 active use days, with an average use of around 50 min per week. The usability measured by SUS yielded a mean score of 74.44 ± 17.46 out of 100 points. Similarly, all domains of the MARS showed values > 3.5 points out of 5, with the highest values in “Functionality”, “Aesthetics” and “Information”. Usability data are demonstrated in Table 2.

**Table 2:** Usability Ratings

All patients (N = 31)	
Active days of App use (up to Follow-Up 1)	49.7 ± 34.6
Average use per week (mins)	50.8 ± 48.8
Average app openings per week	12.0 ± 7.2
SUS (System Usability Score)	74.44 ± 17.46
MARS (Mobile App Rating Scale)	
Engagement	3.67 ± 0.73
Functionality	4.07 ± 0.73
Aesthetics	4.03 ± 0.63
Information	4.00 ± 0.73
Overall Quality	3.94 ± 0.62
Subjective	3.88 ± 0.85

### Patient-reported outcomes

Across the overall cohort (n = 31), changes in patient-reported outcomes were generally small and aligned with the exploratory character of this pilot study.

Fatigue showed a small within-person median increase over 3 months (Hodges–Lehmann Δ =  + 4.0; *r* = 0.16), followed by stable scores at 6 months (Δ =  + 1.0; *r* = 0.12).

Physical quality of life demonstrated the most pronounced improvement, with a median increase by 6 months (Δ =  + 5.3; *r* = 0.50) (see Table 3).

**Table 3:** Patient-reported outcomes in the full cohort (n = 31): non-parametric within-person analysis using Hodges–Lehmann median change and Wilcoxon’s r

All patients, N = 31	Baseline	Follow Up 1 (12 weeks)				Follow Up 2 (24 weeks)			
Measure	(Median[IQR])	(Median[IQR]	Hodges-Lehmann Δ [95% CI]	r	p	(Median[IQR])	Hodges-Lehmann Δ [95% CI]	r	p
Primary outcomes									
FACIT-F	27.0 [21.5–38.0]	31.0 [21.5–39.0]	4.0 (– 1.0 to 3.5)	0.16	0.392	28.0 [22.0–39.0]	1.0 (– 1.5 to 2.5)	0.12	0.530
SF-36 (mental)	46.6 [33.3–53.8]	44.8 [31.5–50.8]	– 1.8 (– 4.3 to 1.2)	– 0.17	0.337	46.7 [35.3–55.4]	0.1 (– 3.0 to 2.6)	– 0.03	0.870
SF-36 (physical)	39.0 [28.1–48.6]	36.8 [29.2–49.6]	– 2.1 (– 0.6 to 2.8)	**0.22**	0.224	44.2 [30.1–50.9]	5.3 (0.9 to 4.6)	**0.50**	**0.005**
Patient pain assessment	5.0 [2.0–6.0]	4.0 [1.0–6.5]	– 1.0 (– 2.0 to 1.0)	– 0.16	0.441	3.0 [2.0–6.5]	– 2.0 (– 2.0 to 0.5)	– 0.25	0.235
Secondary outcomes									
Patient disease activity	4.0 [2.0–7.0]	4.0 [1.5–6.0]	0.0 (– 1.5 to 0.5)	– 0.27	0.196	3.0 [2.0–6.0]	– 1.0 (– 1.5 to 0.0)	**– 0.40**	0.068
Patient Global Assessment	5.0 [2.0–6.0]	4.0 [2.0–5.0]	– 1.0 (– 1.5 to 0.0)	**– 0.56**	**0.020**	4.0 [1.5–5.5]	– 1.0 (– 2.0 to 0.0)	**– 0.51**	**0.039**
HAQ	0.4 [0.0–1.1]	0.4 [0.0–0.8]	0.0 (– 0.3 to 0.0)	**– 0.47**	0.052	0.5 [0.0–0.8]	0.1 (– 0.3 to 0.1)	**– 0.33**	0.145
HLS	36.0 [32.0–43.0]	37.0 [30.0–39.5]	1.0 (– 3.5 to 0.5)	– 0.28	0.126	36.0 [30.0–40.0]	0.0 (– 2.5 to 0.6)	– 0.18	0.317
IPAQ	3918.0 [1452.0–5157.8]	4770.0 [1335.5–6593.2]	852.0 (– 909.8 to 1477.0)	0.09	0.626	3820.0 [2131.5–8718.2]	– 98.0 (– 416.0 to 4468.9)	0.22	0.229
TSK	36.0 [31.0–39.5]	33.0 [27.0–42.0]	– 3.0 (– 4.0 to 0.5)	– 0.22	0.228	34.0 [29.5–40.0]	– 2.0 (– 2.5 to 1.5)	– 0.13	0.500
PAIN-Detect	12.0 [6.5–18.0]	11.0 [7.5–23.0]	– 1.0 (– 0.5 to 3.5)	0.28	0.119	11.0 [5.5–20.0]	– 1.0 (– 2.5 to 1.0)	– 0.11	0.536
PAHCO									
Movement Competence	9.9 [7.8–13.6]	9.3 [7.8–14.4]	– 0.6 (– 0.9 to 0.3)	– 0.09	0.622	11.2 [8.0–12.6]	1.3 (– 0.6 to 0.7)	0.01	0.977
Control Competence	6.4 [5.3–7.9]	6.2 [5.1–7.9]	– 0.3 (– 0.7 to 0.3)	– 0.16	0.375	6.3 [5.0–7.2]	– 0.1 (– 0.7 to 0.4)	– 0.10	0.581
Self Regulation Competence	10.7 [8.8–11.4]	9.6 [8.3–10.6]	– 1.1 (– 1.1 to 0.1)	– 0.28	0.115	10.2 [8.5–11.4]	– 0.5 (– 0.9 to 0.3)	– 0.18	0.327

Note: Values are presented as Median [IQR]. Change estimates represent Hodges–Lehmann (HL) median differences with 95% confidence intervals (CIs). Effect sizes (*r*) are derived from the standardized Wilcoxon statistic (*Z*/√*N*) and indicate the magnitude and direction of within-person change. P-values are reported descriptively, without correction for multiple testing, and are not intended for inferential interpretation given the exploratory nature of this pilot study

*FACIT-F* Functional Assessment of Chronic Illness Therapy–Fatigue; *SF-36* 36-Item Short Form Health Survey; *HAQ* Health Assessment Questionnaire; *HLS* Health Literacy Score; *IPAQ* International Physical Activity Questionnaire; *TSK* Tampa Scale for Kinesiophobia; *PAHCO* Physical Activity–Related Health Competence (movement, control, self-regulation competences)

Bold values indicate HL estimates with p < 0.05 or effect sizes with r > 0.3, reflecting more pronounced within-person changes

Missing *r*- or p-values may occur when fewer than three non-zero paired differences were available or when sample size was insufficient for stable estimation

In contrast, mental quality of life remained largely unchanged throughout the observation period (Δ ≈ 0; *r* < 0.2).

Pain intensity (VRS) decreased modestly from baseline to 3 months (Δ = –1.0; *r* = 0.16) and continued to show small within-person reductions at 6 months (Δ = –2.0; *r* = 0.25).

Other secondary patient-reported outcomes, including functional capacity (HAQ) and health literacy (HLS), remained stable over time with only minimal changes (|Δ|< 1 point; *r* < 0.3) (see Table 4).

**Table 4:** Patient-reported outcomes in the subgroup with disease duration ≤ 2 years (n = 12): non-parametric within-person analysis using Hodges–Lehmann median change and Wilcoxon’s r

RA patients with short disease duration (< = 2 years), N = 12	Baseline	Follow Up 1 (12 weeks)				Follow Up 2 (24 weeks)			
Measure	(Median[IQR])	(Median[IQR]	Hodges-Lehmann Δ [95% CI]	r	p	(Median[IQR])	Hodges-Lehmann Δ [95% CI]	r	p
Primary outcomes									
FACIT-F	26.5 [23.2–30.8]	28.5 [23.5–37.0]	2.0 (– 2.0 to 7.5)	**0.32**	0.311	27.0 [22.0–31.2]	0.5 (– 1.5 to 2.0)	–	–
SF-36 (mental)	40.7 [34.2–49.3]	34.5 [33.4–47.8]	– 6.3 (– 6.7 to 2.6)	– 0.01	0.970	38.4 [34.4–49.5]	– 2.3 (– 5.4 to 4.2)	– 0.03	0.910
SF-36 (physical)	34.3 [29.7–40.0]	35.6 [32.1–46.1]	1.3 (0.5 to 6.7)	**0.67**	**0.021**	43.0 [31.5–47.1]	8.7 (1.1 to 7.5)	**0.85**	**0.003**
Patient pain assessment	5.0 [2.0–6.0]	3.5 [1.8–5.0]	– 1.5 (– 2.5 to 1.5)	– 0.17	0.569	3.5 [2.0–5.2]	– 1.5 (– 2.5 to 1.5)	– 0.17	0.569
Secondary outcomes									
Patient disease activity	5.0 [3.8–7.0]	5.0 [2.8–6.2]	0.0 (– 2.5 to 1.0)	– 0.21	0.512	5.0 [2.8–5.0]	0.0 (– 2.0 to 0.5)	**– 0.37**	0.240
Patient Global Assessment	5.0 [3.5–6.2]	4.0 [2.8–5.0]	– 1.0 (– 2.0 to 0.5)	**– 0.37**	0.240	4.5 [1.8–5.0]	– 0.5 (– 1.5 to 0.5)	**– 0.32**	0.317
HAQ	0.6 [0.3–1.0]	0.4 [0.1–0.7]	– 0.2 (– 0.4 to 0.0)	–	–	0.6 [0.2–0.8]	0.0 (– 0.2 to 0.4)	0.27	0.385
HLS	36.0 [32.2–43.0]	38.0 [30.5–41.2]	2.0 (0.0 to 3.0)	0.14	0.647	36.0 [34.2–40.0]	0.0 (– 2.0 to 3.0)	0.17	0.555
IPAQ	4300.5 [3038.7–5701.1]	5548.5 [2519.2–6703.6]	1248.0 (– 2803.5 to 2700.0)	– 0.05	0.850	6206.5 [3813.0–9522.1]	1906.0 (– 780.0 to 8863.9)	**0.34**	0.233
TSK	38.0 [35.0–41.5]	41.0 [27.8–44.0]	3.0 (– 6.0 to 4.0)	– 0.11	0.715	38.5 [32.0–43.2]	0.5 (– 5.5 to 3.5)	– 0.14	0.649
PAIN-Detect	12.0 [9.2–17.2]	10.0 [8.8–17.2]	– 2.0 (– 3.5 to 3.5)	0.01	0.984	11.0 [6.0–13.0]	– 1.0 (– 4.5 to 1.0)	**– 0.43**	0.140
PAHCO									
Movement Competence	9.8 [9.1–11.6]	9.2 [8.3–10.8]	– 0.7 (– 1.4 to 0.6)	– 0.10	0.733	10.6 [8.4–11.8]	0.7 (– 1.0 to 1.2)	0.03	0.910
Control Competence	6.4 [5.4–8.2]	6.1 [5.3–7.1]	– 0.3 (– 1.5 to 0.3)	**– 0.34**	0.275	6.5 [5.4–7.2]	0.1 (– 0.9 to 0.3)	**– 0.30**	0.301
Self Regulation Competence	10.5 [9.1–11.1]	9.7 [8.6–10.1]	– 0.9 (– 1.8 to 0.5)	– 0.21	0.470	10.3 [8.2–11.0]	– 0.2 (– 1.6 to 0.7)	– 0.24	0.412

Note: Values are presented as Median [IQR]. Change estimates represent Hodges–Lehmann (HL) median differences with 95% confidence intervals (CIs). Effect sizes (*r*) are derived from the standardized Wilcoxon statistic (*Z*/√*N*) and indicate the magnitude and direction of within-person change. P-values are reported descriptively, without correction for multiple testing, and are not intended for inferential interpretation given the exploratory nature of this pilot study

*FACIT-F* Functional Assessment of Chronic Illness Therapy–Fatigue; *SF-36* 36-Item Short Form Health Survey; *HAQ* Health Assessment Questionnaire; *HLS* Health Literacy Score; *IPAQ* International Physical Activity Questionnaire; *TSK* Tampa Scale for Kinesiophobia; *PAHCO* Physical Activity–Related Health Competence (movement, control, self-regulation competences)

Bold values indicate HL estimates with p < 0.05 or effect sizes with r > 0.3, reflecting more pronounced within-person changes

Missing *r*- or p-values may occur when fewer than three non-zero paired differences were available or when sample size was insufficient for stable estimation

In the subgroup of recently diagnosed patients (≤ 2 years; n = 12), changes were directionally consistent but more pronounced.

Physical quality of life improved at both follow-up assessments (Δ₃ₘ =  + 1.3; *r* = 0.67; Δ₆ₘ =  + 8.7; *r* = 0.85), indicating a large within-person effect.

Fatigue and pain also improved numerically (FACIT-F Δ =  + 2.0; *r* = 0.32; pain Δ = – 1.5; *r* = 0.17), whereas mental quality of life remained stable.

### Qualitative results

To better identify which patient group benefits from the digital health application and which may experience barriers to use, qualitative feedback from the follow-up telephone check-ins was clustered into four user personas.

The four user profiles were developed inductively based on patterns identified in participants´ follow-up interviews. Notes from the structured telephone calls were summarized in an Excel spreadsheet and grouped according to recurring themes and user characteristics (e.g. age, disease activity, time of diagnosis, coping style). The clustering process by two independent researcher (VS, HM) was conducted descriptively to illustrate typical user experiences rather than to perform a formal qualitative analysis.

The quotes were selected to represent the main themes and to exemplify each user profile. Selection was based on clarity, relevance and typicality of the statements rather than systematic coding or thematic saturation. Additional quotes exemplifying each one of the clustered user groups can be found in the Supplementary Material (Supplement Quotes). 

#### Type 1: stable condition with established routines

This group includes mostly younger patients or those with well-controlled symptoms and stable disease activity. In many cases they tend to have existing routines for physical activity and symptom tracking, and already developed effective coping strategies. They therefore use the app occasionally or primarily as a supplementary tool.

##### Participant A, 24 years

*"Due to my well-managed medication regimen, my symptoms are currently very stable. Therefore, symptom tracking is no longer as relevant for me. Additionally, as I am currently pain-free, I engage in a lot of physical activity and go to the gym, so the exercise videos offered in the app are more of a nice extra. However, I can well imagine that the app is particularly helpful for newly diagnosed patients. For example, it would certainly have supported me in the time shortly after my initial diagnosis."*

#### Type 2: patients with low engagement due to avoidant coping or high disease activity

Patients in this group tend to use the app minimally, benefiting little from its self-management features. Reasons for this can include strong physical and psychological strain due to active symptoms such as pain, fatigue, and stress. During these phases, the capacity for structured self-management and the energy for additional health-related tasks is reduced. As a result, digital interventions may be perceived as too cognitively or practically demanding. The app can feel overwhelming and doesn’t fit well into their current routines, even if it could help in the long run. Another subgroup within this type include patients who express a desire to keep their focus away from the disease. For them, engaging with disease-related content such as reflection questions or educational content can feel emotionally draining or intimidating.

##### Participant C, 55 years

*"For me personally, focusing so much on the disease can also be straining. I currently want to keep my mind more free from the illness. Therefore, I am not the type to engage much with reflection questions or the educational course."*

#### Type 3: experienced patients with analog habits and openness to new approaches

This group consists of patients with long-term disease experience who often primarily used analog methods such as paper-based symptom tracking. Despite some having initial difficulties with the app’s usabilty due to limited technical familiarity, they demonstrate openness to digital tools – but as additional support rather than their main tool. They value personal interactions and their established habits alongside new approaches and often find specific features helpful, especially those adapted to physical limitations.

##### Participant E, 68 years

*"Symptom tracking helps me a lot. I can see how my condition changes throughout the day and week. For example, I noticed how sensitive I am to weather changes."*

#### Type 4: newly diagnosed or highly engaged patients seeking structure and guidance

This group includes patients with a short disease duration or those actively seeking strategies to cope with their symptoms. They are often highly motivated to understand their condition and integrate supportive behaviors into everyday life. For them, the app serves as a source for orientation, knowledge, and educational content—particularly during the early or unstable stages of disease management.

##### Participant H, 60 years

*"The gymnastics exercises motivate me—I often do them together with my husband and grandson. The educational course is also very helpful. For example -the information that symptoms like sleep disturbances and hot flashes can be related to rheumatoid arthritis was new to me."*

## Discussion

This exploratory mixed-methods feasibility study primarily assessed usability and adherence, and secondarily explored trends in patient-reported outcomes of a DHA for patients with RA. Overall, usability ratings were high, and adherence to the app—especially to its core components such as the daily schedule, symptom tracking, and physical exercises—was good. As already demonstrated in other recent publications, this study shows that digital technologies for rheumatic diseases are necessary in this field of medicine and are well accepted by users [38, 39]. In a Germany-wide survey of rheumatology patients with the majority diagnosed with RA, the greatest number of patients (72.4%) would like to use a digital health application and would be happy to receive recommendations from the attending rheumatologist (72.8). 76.0% would prefer a DHA with rheumatological focus [40]. However, adherence and compliance are a significant challenge with digital technologies [41, 42]. As the long-term effects of education and physical activity often only appear relatively late, it is particularly important that compliance is maintained. Our study shows that usage decreases over the course of the study. Options for ensuring long-term use could include telephone check-ins, reminders or gamification in the area of exercise therapy. Moreover, digital interventions are rather new in medicine and it makes sense to address the concept of adherence more closely. There is often a lack of the right measurement parameters and one needs to move away from the view that more intensive use of digital interventions automatically leads to better results [43].

In our study, changes in clinical and patient-reported outcomes across the total sample were small, with most effect sizes in the negligible to small range. These findings should be interpreted in light of the study population, which consisted predominantly of patients with a long-standing RA (mean disease duration: 5.3 years). It is well established that patients with a longer disease history may already have developed individualized coping strategies, established routines, and stable treatment regimens. As a result, they may exhibit lower responsiveness to new interventions, particularly when such interventions target behavior changes or self-management. Additionally, patients with long-standing RA may show lower motivation to adopt new habits or technologies, especially if their symptoms are perceived as manageable. This pattern aligns with behavioral models of health behavior change, which suggest that perceived need and openness to new interventions tend to be higher earlier in the disease trajectory, when uncertainty, emotional distress, and informational needs are more pronounced [44].

Consistent with the overall exploratory findings, subgroup analyses indicated that patients with shorter disease duration (≤ 2 years) showed more pronounced within-person improvements across several patient-reported outcomes, particularly in physical quality of life and fatigue. These changes corresponded to small-to-moderate effect sizes and exceeded those observed in the overall cohort. This pattern suggests that digital self-management interventions may be especially beneficial during the early phase of rheumatoid arthritis, when patients are still adapting to the diagnosis, developing coping strategies, and building disease-specific competencies. The qualitative findings further support this interpretation, revealing that highly engaged and motivated users were frequently those with recently diagnosed disease, who actively sought structure, guidance, and self-management support through the app.

These findings are in accordance with previous work showing that the timing of educational or behavioral interventions plays a critical role in their effectiveness. For instance, early intervention programs in chronic diseases have shown higher uptake, stronger behavioral engagement, and more substantial long-term benefits compared to interventions introduced later in the disease course [45, 46]. The beginning of the patient journey can thus be seen as a "window of opportunity" for establishing positive health behaviors, promoting self-efficacy, and preventing long-term disability [47].

Despite the overall positive trends, no improvement was observed in health literacy (HLS), and a slight deterioration was noted in physical activity-related competencies (PAHCO), particularly RA patients with shorter disease duration. It is particularly interesting that the PAHCO improves the competence of self-regulation both in patients in general and in patients with a shorter diagnosis. As the founders of the questionnaire have already described, this concerns the motivation to plan and carry out sports. As the sports exercises of the app in particular were used frequently, these results appear to be consistent with the objective user analysis [48]. The lack of change in health literacy is in fact not surprising. It may be reflected by the short duration of the intervention and the limited intensity of content specifically targeting these domains. Alternatively, the increased awareness resulting from self-monitoring and app-based education could have temporarily led patients to reassess their physical activity skills more critically—a phenomenon sometimes referred to as a “response shift” [49]. Future iterations of the app may benefit from more explicit content on health literacy and competence-building strategies in the context of physical activity, such as goal setting, and self-efficacy enhancement.

Considering long-term effects of the intervention, the 6-month follow-up period indicated potential sustained stabilizing effects for certain outcomes This suggests that early intervention in this subgroup may contribute to longer-lasting benefits. The literature supports that exercise and educational interventions often require extended periods to exert their full impact on parameters like pain, quality of life, and global impairment. For example, long-term exercise training exceeding 12 weeks has been shown to reduce disease activity and clinical severity and may be particularly beneficial in patients with early stages of RA, highlighting the value of early implementation in treatment plans [50]. Similarly, patient education and self-management programs may show limited short-term effects but contribute to meaningful benefits over longer durations. Previous research indicates that improvements after self-management training may not become evident until several months after the intervention [51]. Nevertheless, some studies also suggest that the change that educational or self-management resources for patients of chronic diseases can be sustained over extended periods of time [52–54]. Therefore, the app-based combination of physical activity and educational resources used in this study may also need longer follow-up to demonstrate the full spectrum of benefits, especially regarding possible long-term effects like pain reduction and better quality of life.

Several limitations should be acknowledged. First, the pilot design without a control group and the small sample size limits the generalizability of the findings and preclude causal conclusions.

Without a control group, the observed effects cannot be attributed solely to the use of the app, but may also reflect the influence of other confounding variables. Possible confounders include changes in medication, disease activity or general health decline due to other illnesses or comorbid conditions. Moreover, general lifestyle changes outside of the app use such as exercise, dietary changes or participation in other therapies may be contributing factors to the findings of our study.

Second, all outcome measures were self-reported, which may be subject to recall or response bias. Third, the follow-up duration of 12 weeks for the first, and 24 weeks for the second assessment may have been too short to capture long-term behavioral changes or sustained clinical improvements, especially when it comes to health competency, physical activity related health competency and fear of motion. Also changes in the physical activity behavior are long-term effects and usually don´t happen after a short period of exercising. The qualitative analysis was not intended to achieve saturation but to contextualize quantitative findings and illustrate typical user experiences. Additionally, the sample consisted mainly of patients with long-standing RA, which may have influenced responsiveness. Finally, subgroup analyses were exploratory and based on limited numbers, warranting cautious interpretation. All results should be interpreted as exploratory signals informing the design of future randomised controlled trials.

In summary, this exploratory feasibility study demonstrates that the Fimo Health App can provide low-threshold, scalable digital support for patients with rheumatoid arthritis. Within-person analyses indicated small-to-moderate improvements in physical quality of life, fatigue, and pain, particularly among patients with shorter disease duration. These findings emphasize the importance of tailoring digital self-management interventions to individual patient profiles and stages of disease progression.

Given the uncontrolled study design, the observed changes cannot be interpreted causally and may also reflect natural fluctuations or non-specific effects. Nevertheless, the results provide valuable insights for the iterative refinement of the intervention, highlighting the need to enhance engagement with the structured course materials—potentially through adaptive content delivery, gamification, or peer-support features.

Future randomized controlled trials are warranted to validate these exploratory findings and to determine the app’s clinical effectiveness under controlled conditions.

## Supplementary Information

Below is the link to the electronic supplementary material.

Supplementary file1 (DOCX 393 KB)

## Data Availability

The data sets are available on reasonable request from the corresponding author.
