# Supplementary material for: Evaluating usability, adherence and clinical benefit of a new digital heath application in rheumatoid arthritis: a pilot feasibility study
Source: Rheumatol Int. 2026 Jan 10;46(1):30. doi: 10.1007/s00296-025-06060-6 (PMC12790543; doi:10.1007/s00296-025-06060-6)
Supplement: Supplementary file 1 — Supplementary file1 (DOCX 393 KB) [file 296_2025_6060_MOESM1_ESM.docx]

**
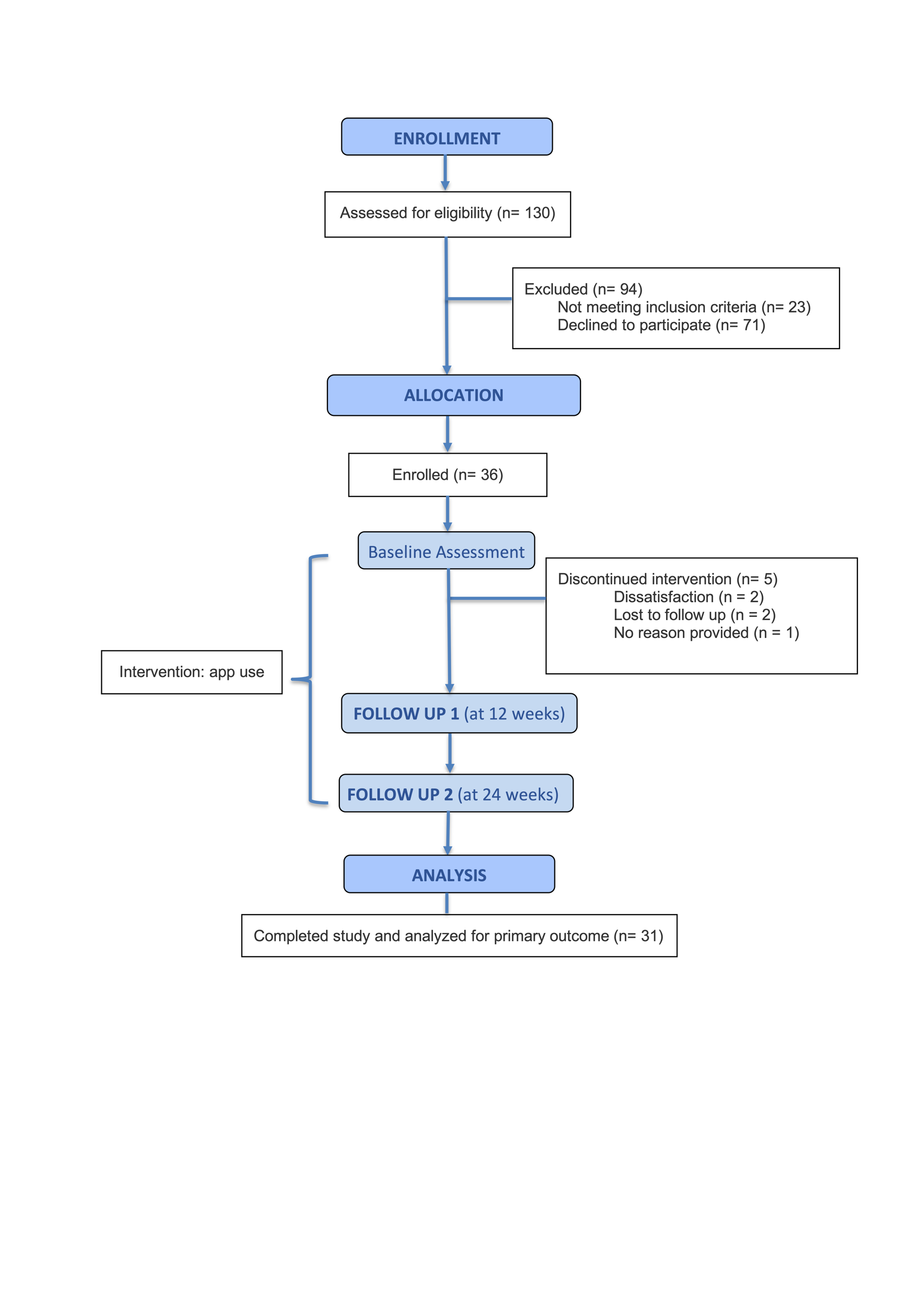
**

**Supplement Figure 2: Patient Flowchart**

**Supplement Figure 3: Chronological overview of study timeline and assessments**

**Supplement Table 1:** Patient reported outcomes

| Questionnaires | Abbreviation | Unit | Range | Measurement | References |
| --- | --- | --- | --- | --- | --- |
| Verbal Rating Scale | VRS | Scale | 0 to 10:  0 = no pain,  10 = highest pain | Patient perceived pain in the last 7 days, generally and especially back pain | [25] |
| Pain Detect | PAIN | Score | 0 to 38:  0-12 = neuropathic pain component unlikely,  13-18 = uncertain,  19-38 = neuropathic pain component probably | Detection of neuropathic pain | [26] |
| Tampa Scale of Kinesiophobia | TSK | Score | 17 to 68:  17-37 = low degree of kinesiophobia,  37-68 = high degree of kinesiophobia | Fear of movement | [27] |
| Physical Activity-related Health Competence | PAHCO | Total  Mean  Percent | Individual  0-10  0%-100% | Physical activity related skills: movement competence, control competence, self-regulation competence | [28] |
| Health Assessment Questionnaire | HAQ | Score | 0 to 3:  0-1 = mild to moderate disability,  1-2 = moderate to severe disability,  2-3 = severe to very severe disability | Extent of limitations in physical function | [29] |
| Health Competence | HLS-EU-Q16 | Score | 16 to 64:  Higher score indicates a better health competence | Health competence, measured by 16 items | [30] |
| Short Form 36 Questionnaire | SF-36 | Score | 0 to 100:  higher score indicates a better health status | Health-related quality of life, 36 items that assess physical and psychological health | [31] |
| Work Productivity and Activity Impairment | WPAI | Percentage | 0% to 100%:  higher percent means less work productivity and greater impairment | Effects of the chronic illness on the ability to work and the daily activities | [32] |
| International Physical Activity Score | IPAQ | Score |  | Measurement of daily activity | [33] |

**Supplement Quotes:** Additional quotes for each user profile

**Type 1: Stable condition with established routines**

***Participant B, 53 years:*** *"I have been living with rheumatoid arthritis for quite some time and have been tracking my symptoms on paper for several years. My medications are firmly scheduled in my personal calendar. Also, due to my medical background, I was already familiar with most of the information in the educational course. Therefore, I only use the app occasionally — personal exchange with other patients is more important to me."*

**Type 2: Patients with low engagement due to avoidant coping or high disease activity**

**Participant D, 45 years:**
"Due to the disease and stress, I currently have little motivation. The app is somewhat inconvenient for me and, in my current condition with pain and fatigue, rather an additional burden."

**Type 3: Experienced patients with analog habits and openness to new approaches**

**Participant F, 40 years:**
"The stretching exercises and yoga are very well-designed. I notice they help with my back problems. Overall, I find the app concept very thoughtful.

**Participant G, 71 years:**
"I had to get used to the technology first - luckily, my grandson helped me. Some of the exercises were not feasible for me due to knee problems. Therefore, I especially liked the seated-yoga and stretching exercises."

**Type 4: Newly diagnosed or highly engaged patients seeking structure and guidance**
This group includes patients with a short disease duration or those actively seeking strategies to cope with their symptoms. They are often highly motivated to understand their condition and integrate supportive behaviors into everyday life. For them, the app serves as a source for orientation, knowledge, and educational content —particularly during the early or unstable stages of disease management.

**Participant I, 67 years:**
"The app complements the information provided by doctors well. In clinical consultations, there is often little time for detailed discussions — the educational course helps here. Through the symptom descriptions, patients feel directly acknowledged and understood."

**Participant J, 55 years:**
"The meditations and sound journeys help me with pain, and the relaxation exercises before bedtime helped improve my sleep to some extent. Fatigue is the main symptom I struggle with, and in my opinion, the app is very well suited to address this. “
